# Pollution transfer and environmental health implications: network evolution and proximity mechanisms in the Yangtze River Delta, China

**DOI:** 10.3389/fpubh.2026.1770901

**Published:** 2026-02-16

**Authors:** Feng Hu, Huijie Yang, Xiaolong Zhou, Shuang Zhao, Liping Qiu, Shaobin Wei, Xiaoping Wang, Jiahan Hu, Yufeng Chen, Hao Hu, Haiyan Zhou

**Affiliations:** 1 Institute of International Business & Economics Innovation and Governance, Shanghai University of International Business and Economics, Shanghai, China; 2 Industrial Technology Research Center, Shanghai Yice Research Institute, Shanghai, China; 3 International Business School, Shanghai University of International Business and Economics, Shanghai, China; 4 School of Law, Shanghai University of International Business and Economics, Shanghai, China; 5 College of Business Administration, Ningbo University of Finance and Economics, Ningbo, China; 6 CEEC Economic and Trade Cooperation Institute, Ningbo University, Ningbo, China; 7 College of Engineering, University of Perpetual Help System Laguna, Biñan, Laguna, Philippines; 8 School of Economics and Management, Zhejiang Normal University, Jinhua, China; 9 School of Economics, Shanghai University, Shanghai, China; 10 Graduate School, Nueva Ecija University of Science and Technology, Cabanatuan, Philippines

**Keywords:** environmental health risk, Geodetector, pollution transfer, proximity mechanisms, public health proximity, social network analysis, Yangtze River Delta

## Abstract

Pollution transfer across regions has become an important source of environmental health risks and spatial health inequalities, yet its underlying transfer mechanisms and structural pathways remain insufficiently understood. This study investigates the spatiotemporal evolution of pollution transfer in China’s Yangtze River Delta (YRD) from both national and local perspectives. Using off-site environmental penalty data from large-scale enterprises between 2012 and 2023, we construct pollution transfer networks and apply social network analysis and the geographical detector method to identify structural characteristics and multidimensional driving mechanisms. This study addresses three interrelated challenges: identifying pollution transfer relationships from regulatory records, disentangling multidimensional proximity mechanisms and their interaction effects, and assessing whether pollution transfer structurally reinforces environmental health inequality across cities, which are tasks that are methodologically demanding due to the hidden nature of transfer processes and the nonlinearity of proximity effects. The results reveal that the pollution transfer network in the YRD has continuously expanded, exhibiting a pronounced core–periphery structure centered on Shanghai, Hangzhou, Nanjing, and other core cities, with strong internal linkages and outward diffusion at the national scale. Pollution transfer intensity has increased over time and displays significant proximity effects. Multidimensional proximity factors jointly shape pollution transfer, with economic proximity dominating intra-regional transfers, while institutional and technological proximity play a more critical role in extra-regional transfers. Notably, public health proximity exhibits limited independent explanatory power but significantly enhances pollution transfer when interacting with economic and institutional factors, suggesting a structural linkage between pollution redistribution and environmental health inequality. Scientifically, this study advances the understanding of pollution transfer as a networked and interaction-driven process. Practically, the findings provide quantitative support for regional collaborative governance and integrated environmental and health management in the YRD.

## 1 Introduction

In recent years, off-site pollution transfer has emerged as a prominent challenge in China’s ecological and environmental governance, with profound implications for environmental health risks and regional health equity. These transfer activities not only have covert and widespread harmful effects but also pose severe threats to regional ecological safety and public health. Research indicates that as local environmental oversight intensifies, enterprises increasingly resort to pollution transfer to mitigate the production costs incurred from environmental penalties (1, 2). Statistical analyses of multiple batches of typical ecological and environmental enforcement cases published by the Ministry of Natural Resources reveal that pollution transfer cases that originate from the Yangtze River Delta (YRD) account for a significant proportion of cross–provincial and cross–municipal cases^1^. This result indicates that the region has become both a major source for and a heavily impacted area by off-site pollution transfer during its rapid industrialization and urbanization. Consequently, research on pollution transfer in the YRD holds critical significance for addressing pollution transfer governance nationwide.

Studies on pollution transfer originated with Walter’s “pollution haven hypothesis,” which posits that polluting industries migrate from developed to relatively underdeveloped countries and transform underdeveloped countries into “paradises for pollution transfer (69)”. Building on this theoretical foundation, a substantial body of empirical research has examined the relationships between environmental pollution and international trade, foreign direct investment, environmental regulation, industrial policy, and firms’ location choices. For example, Cole and Elliott find that stringent environmental regulations in developed economies tend to induce the relocation of pollution-intensive industries to regions with weaker regulatory enforcement, providing empirical support for the pollution haven hypothesis (3). Similarly, studies by Wang et al. (4) and Wu et al. (5) demonstrate that heterogeneity in regulatory stringency plays a significant role in reshaping the spatial distribution of polluting industries. However, a growing number of studies have challenged the simplistic interpretation of the pollution haven hypothesis. Ramanathan et al. (6) argue that well-designed environmental regulations can stimulate technological innovation and efficiency improvements, thereby generating an “innovation compensation effect”. Focusing on multinational gold-mining firms, Tole and Koop (7) show that firms’ relocation decisions are also constrained by relocation costs, capital advantages, and sunk investments, implying that stricter regulations do not automatically trigger pollution transfer. Moreover, drawing on empirical evidence from heavy-polluting industries in China, Zhang et al. (8) find that environmental regulation can promote green production and technological innovation by leveraging regional comparative advantages in factor endowments, while simultaneously balancing market resource allocation and cost-correction mechanisms, thus contributing to economic development. Taken together, these studies indicate that the relationships among regulatory stringency, industrial characteristics, and pollution transfer are nonlinear and highly context-dependent, with industrial policy increasingly recognized as a key mediating factor shaping the dynamic evolution of pollution transfer processes (3–19).

Pollution transfer studies rely primarily on metrics such as industrial location entropy, carbon emission intensity changes, the number of polluting enterprises, the share of polluting industries in Gross Domestic Product (GDP), and the parent–subsidiary links of polluting firms. Collectively, this body of research reveals that pollution transfer is not a simple outcome of regulatory differentials, but a multidimensional, nonlinear, and structurally embedded process shaped by the interplay of regulatory, social, and network mechanisms (20–23, 70). However, these approaches exhibit significant measurement errors when estimating pollution transfer patterns and therefore fail to reflect actual pollution transfer dynamics accurately. With the maturation of big data collection techniques, scholars have begun to explore environmental penalty records to study corporate pollution behavior (24–28). Current research, however, predominantly utilizes penalty data to evaluate regional environmental regulatory efficiency or corporate environmental performance. For instance, Liao’s study analyzes solely from the perspective of corporate behavior how public demands significantly promote green investment by strengthening local government environmental regulations (25). It has yet to fully explore the inherent spatial patterns of pollution transfer embedded within this process.

As national attention to pollution transfer intensifies and environmental regulations tighten across regions, many cities have witnessed spatially directed pollution transfer disguised as industrial relocation. Analyzing pollution transfer through an urban network perspective has significant policy implications for promoting coordinated regional pollution governance. Urban network studies rely primarily on data streams from Baidu searches or social media, patent cooperation and transfer, paper collaboration flows, train schedules, or Baidu migration population flows, and ownership relationship data from an enterprise perspective. Alternatively, they employ gravity models based on cities’ economic attributes to estimate inter–city connection strengths or use social network analysis methods to calculate network metrics such as centrality and clustering coefficients, thereby revealing spatial heterogeneity and core–periphery structures (29–42). With respect to urban network influencing factors, scholars have analyzed network impact mechanisms using quadratic assignment problem (QAP) models, negative binomial regressions, exponential family random graph models, and geographic detectors (43–45). In recent years, the multidimensional proximity mechanisms of network structure—including geographic, institutional, economic, and industrial dimensions—have attracted significant attention. The interactions between different proximity types have emerged as a research hotspot in urban network influencing mechanisms (46–50). Boschma (48) argues that proximity can address coordination and lock-in problems either individually or in combination. Multidimensional proximity influences actors’ coordination and performance through processes of mutual substitution and complementarity, thereby providing a critical theoretical foundation for analyzing urban network formation from an interaction-effect perspective. Building on this framework, empirical studies by Chinese scholars, such as Cao et al., (49) further demonstrate that interaction effects among different proximity dimensions are often more influential than single-factor effects, making proximity interactions a key frontier for explaining the evolution of urban networks and their spatial environmental linkages in the Chinese context.

As one of China’s most economically dynamic regions, the YRD faces substantial ecological and environmental pressures, making it a representative case for investigating pollution transfer and its public health implications. By the end of 2018, the integration of the YRD was elevated to a national strategy, with “joint ecological and environmental protection and governance” as a core component. Given this context, this study analyzes the structural characteristics and proximity mechanisms of the pollution transfer network in the YRD from a dual national–local perspective, with explicit attention to environmental health risks. This study aims to address the following key scientific questions: (1) Is pollution transfer among cities in the YRD a networked and structurally asymmetric process rather than a random or bilateral relocation phenomenon? (2) Which dimensions of proximity, geographic, economic, institutional, technological, or public health, drive pollution transfer, and do their interaction effects outweigh single-factor influences? (3) Does pollution transfer reinforce environmental health inequality by redistributing environmental risks toward cities with similar or weaker public health capacities? Addressing these questions is methodologically demanding because pollution transfer is latent, proximity effects are multidimensional and interactive, and environmental health inequality is structurally embedded rather than directly observable.

This study provides a theoretical foundation and practical reference for national policy formulation, regional collaborative pollution control, and public health management. It enriches the existing literature through three key contributions. To begin with, by employing a dual “national-local” analytical framework to compare pollution transfer network indicators across different scales, it reveals the dynamic characteristics of pollution transfer in the spatial dimension. This approach overcomes the limitations of single-scale research and offers a new hierarchical research paradigm for urban network studies (29–41). Moreover, by leveraging big data on environmental fines imposed on enterprises across diverse regions, this study overcomes the inherent underestimation issues inherent in traditional pollution transfer research based on estimation methods, establishing a new empirical foundation for pollution transfer studies (20–28). Finally, by introducing multidimensional proximity theory, this research systematically reveals the interactive mechanisms among economic proximity, institutional proximity, technological proximity, geographic proximity, industrial proximity, and public health proximity in pollution transfer. Particularly, the interactions between public health proximity and other factors provide a theoretical foundation and policy reference for pollution control and public health management in the YRD and across China (44–50).

## 2 Research data and methods

### 2.1 Research subjects and data

The data referenced in this paper originated from the Qixin Huiyan database. By leveraging off-site environmental penalty records, this study identifies pollution transfer incidents formally disclosed by environmental regulatory authorities. Compared to emission inventories or inferred migration pathways, this data source directly addresses the challenge of observing covert pollution transfer behaviors, a critical approach for investigating pollution control issues in the YRD, characterized by stringent regulation and industrial density. Initially, the “Batch Customer Acquisition” section was utilized to export a list of 45,995 enterprises registered in the YRD with environmental penalty records. Subsequently, the “Batch Query” section was employed to extract environmental penalty module data from the operational risk section of the list, yielding a total of 106,050 entries. Data cleaning and verification were subsequently conducted. Missing or unclear penalty authority and penalty date fields were supplemented manually. Unambiguously unclear data points were deleted. For instance, where the environmental penalty authority was listed as “Municipal Environmental Protection Bureau,” “District Environmental Protection Bureau,” or “County Environmental Protection Bureau,” the Specific County or city of the authority was clarified by cross–referencing the penalty document numbers, penalty descriptions, and legal bases. Then, simple penalty frequency statistics cannot adequately measure the severity of pollution transfer or environmental risk levels. Penalty amounts typically correlate positively with the severity of environmental violations, but this relationship is not strictly linear. Therefore, penalties were weighted based on their monetary value: penalties of 0 ten thousand yuan, 0.01–0.99 ten thousand yuan, 1–9.99 ten thousand yuan, 10–19.99 ten thousand yuan, 20–49.99 ten thousand yuan, 50–99.99 ten thousand yuan, and over 100 ten thousand yuan were assigned weights of 0.5, 1, 1.5, 2, 3, 4, and 5, respectively. Furthermore, the 18th Chinese Communist Party (CCP) National Congress in 2012 first dedicated a separate chapter to “ecological civilization” and incorporated ecological civilization construction into the overall layout of socialism with Chinese characteristics. In the same year, the Ambient Air Quality Standards (GB3095-2012) were introduced. Since 2016, the central government has elevated environmental protection to unprecedented levels through environmental inspections. By late 2018, YRD integration was raised to a national strategy, with “joint ecological and environmental protection and governance” as its core component, which marked a new phase in regional environmental governance collaboration. In 2020, with the introduction of the “dual carbon” goals and the full implementation of the YRD Regional Integration Development Plan Outline, achieving a comprehensive green transformation of economic and social development became a core task. To objectively depict the spatial evolution of pollution transfer in the YRD while avoiding the abrupt changes caused by exceptional circumstances in specific years and considering key policy implementation milestones, this study employs a segmented analysis based on the year that environmental penalties were issued. The research period is divided into three equal segments: 2012–2015, 2016–2019, and 2020–2023. Following these procedures, by querying the Qixin Huiyan database, we identified prefecture–level cities where both regulated enterprises and penalizing authorities were registered. After duplicate environmental penalty records for the same city were removed, the final dataset comprised 18,143 entries from 2012 to 2023. On the basis of the prefecture–level cities of both the enterprises and the penalizing authorities, along with the penalty amounts, we constructed a pollution transfer network across the YRD.

### 2.2 Research methods

From a methodological perspective, this study proposes a problem-oriented analytical framework that integrates regulatory pollution transfer data with network analysis and interaction detection. This approach aims to uncover the concealment of pollution transfers and to identify structural and mechanistic patterns unique to the YRD, thereby providing actionable recommendations for public health governance.

#### 2.2.1 Social network analysis method

Using Gephi software, we analyzed the network density, average degree, and city–weighted centrality of the pollution transfer network in the YRD. This enabled an in-depth examination of the network’s fundamental attributes and node characteristics to understand the spatial patterns and systematic evolution properties of the pollution transfer network (51–54). By modeling pollution transfer as a directed weighted network, this study overcomes the limitations of bilateral and unidirectional analyses, revealing structural patterns in pollution redistribution, such as core-periphery differentiation and asymmetric transfer pathways, that reflect the hierarchical organizational structure within the YRD city cluster.

#### 2.2.2 Geodetector

The factor detection and interaction detection modules of Geodetector software were employed to analyze in depth the relationship between pollution transfer and multidimensional proximity, thereby elucidating the multidimensional proximity mechanisms and revealing system coupling drivers within the pollution transfer network of the YRD (55–59). The application of Geodetector methods can capture effects identified by traditional regression approaches. Particularly when incorporating public health proximity indicators, it enables explicit testing of whether pollution transfer exhibits structural associations with similarities in health carrying capacity, thereby linking pollution redistribution to environmental health inequalities at the inter-city level and revealing how environmental risks are structurally redistributed among cities with similar health capacities in the YRD. Additionally, by explicitly incorporating public health proximity indicators into network mechanism analysis, this study extends traditional geographic network analysis to an environmental public health perspective. This enables the assessment of the relationship between pollution transfer and urban health risks at the urban structural level.

## 3 Results and analysis

### 3.1 Structural characteristics of the overall pollution transfer network from a national–local perspective

This section aims to reveal the overall structural patterns and evolutionary trajectories of pollution transfer centered on the YRD from a national-local perspective, providing a macrofoundation for subsequent network mechanism analysis.

**Figure 1:**
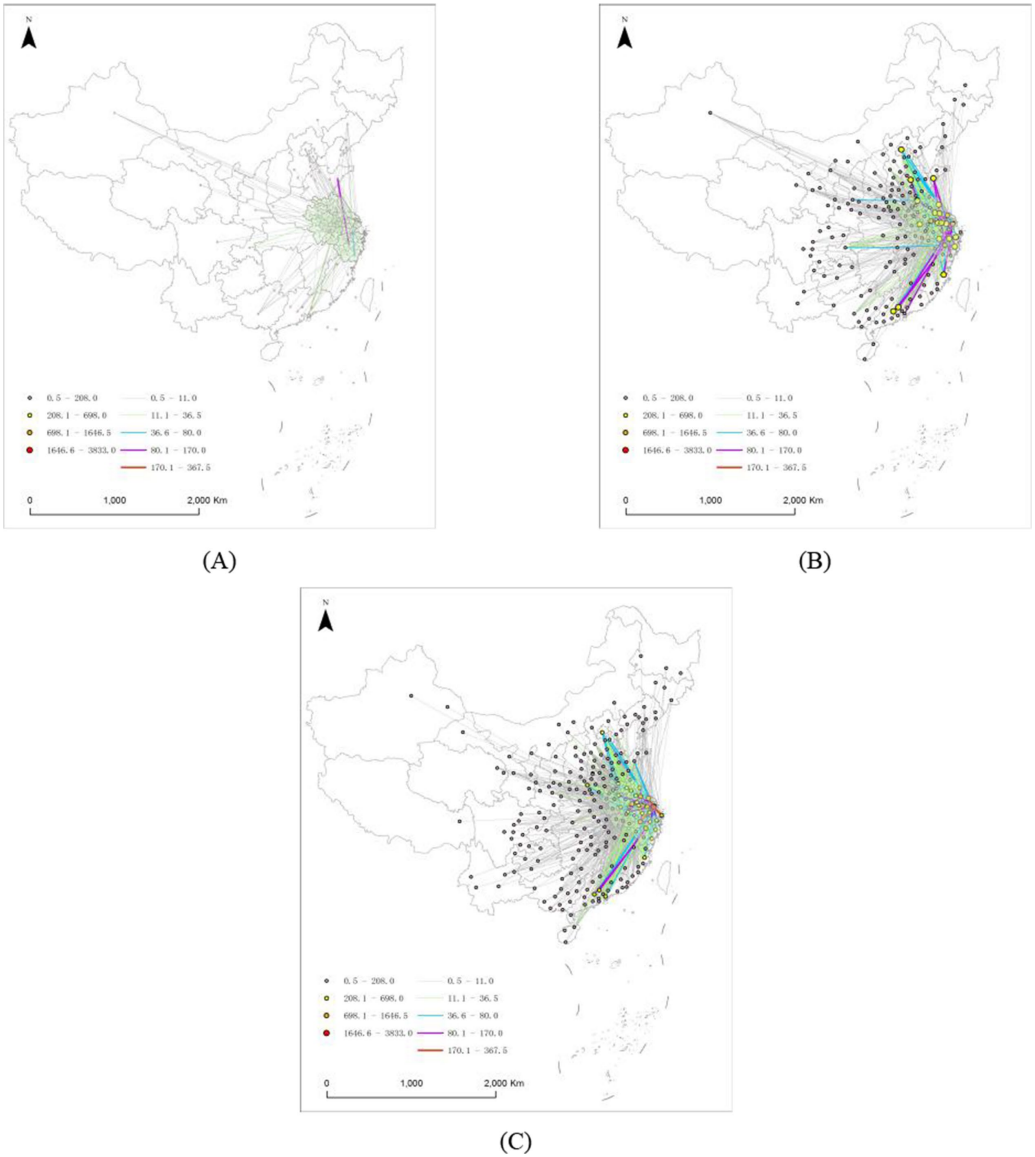
Overall pollution transfer network in the YRD. **(A)** 2012–2015; **(B)** 2016–2019; **(C)** 2020–2023.

The overall pollution transfer network in the YRD continues to expand, with an increasing number of cities involved in cross–city pollution associations and tighter pollution connections between cities (Figure 1). Pollution transfer relationships in the YRD are spatially widespread, covering multiple cities nationwide, forming a complex structure centered on the YRD and radiating across the country. Pollution transfer connections among cities within the YRD dominate and carry higher weights. The number and intensity of inward pollution transfer links within the YRD increased from 77 and 320 in Phase I to 332 and 2,292 in Phase II and further to 496 and 5,933 in Phase III. Moreover, the number and intensity of outward pollution transfers from the YRD were 82 and 359,529, 3,681 and 953, 4,935, respectively. This finding indicates that in Phase I, the depth and breadth of pollution transfer both within and outside the region were roughly comparable. Beginning in Phase II, however, the region gradually shifted toward transferring pollution to cities outside the YRD, increasing the breadth but decreasing the depth of inward transfers. Higher–level pollution connections were concentrated among the core cities within the region, and the intensity of pollution transfer increased. The network exhibits a distinct “core–periphery” structure and forms a spatial configuration with Shanghai at the core and other cities as peripheral nodes, which reveals pronounced urban hierarchy disparities. Compared with existing research, this study transcends the narrow perspective that focuses solely on the spatial correlation and spillover effects of environmental quality between cities (60). Existing studies primarily capture average spillover effects, with limited understanding of the specific pathways, directionality, and structural asymmetries of pollution redistribution. In contrast, this study reveals how cross-city pollution redistribution forms structural patterns such as core-periphery disparities, rather than merely reflecting spatial proximity effects.

### 3.2 Structural characteristics of the YRD’S outward pollution transfer network from a national perspective

Based on the overall network structure, this section focuses on analyzing the external pollution transfer pathways to examine how the YRD interacts with other domestic regions and how these interactions evolve. To further analyze the structural characteristics of the YRD’s outward transfer network, we isolated the interregional pollution transfer network from the overall pollution transfer network. The findings (Table 1) reveal that the number of cities participating in the YRD’s outward pollution transfer increased from 59 to 229, the number of pollution transfer edges rose from 91 to 1,090, and the average degree and average weighted degree increased from 1.542 and 6.161 to 4.760 and 21.849, respectively. The network diameter and average path length both reached 1, while the average clustering coefficient remained at 0. The modularity index decreased from 0.479 to 0.271. These results indicate that the scale of the YRD’s outward pollution transfer network continues to expand, with both the volume and intensity of pollution transfers increasing. The network exhibits high connectivity, and pollution transfer activities are increasingly characterized by off-site movement.

**Table 1:** Basic attributes of outward pollution transfer networks from the YRD.

Statistical indicators	Phase I	Phase II	Phase III
Node	59	170	229
Edge	91	616	1,090
Average degree	1.542	3.624	4.76
Average weighted degree	6.161	21.912	21.849
Network diameter	1	1	1
Density	0.027	0.021	0.021
Modularity	0.479	0.349	0.271
Average clustering coefficient	0	0	0
Average path length	1	1	1

**Figure 2:**
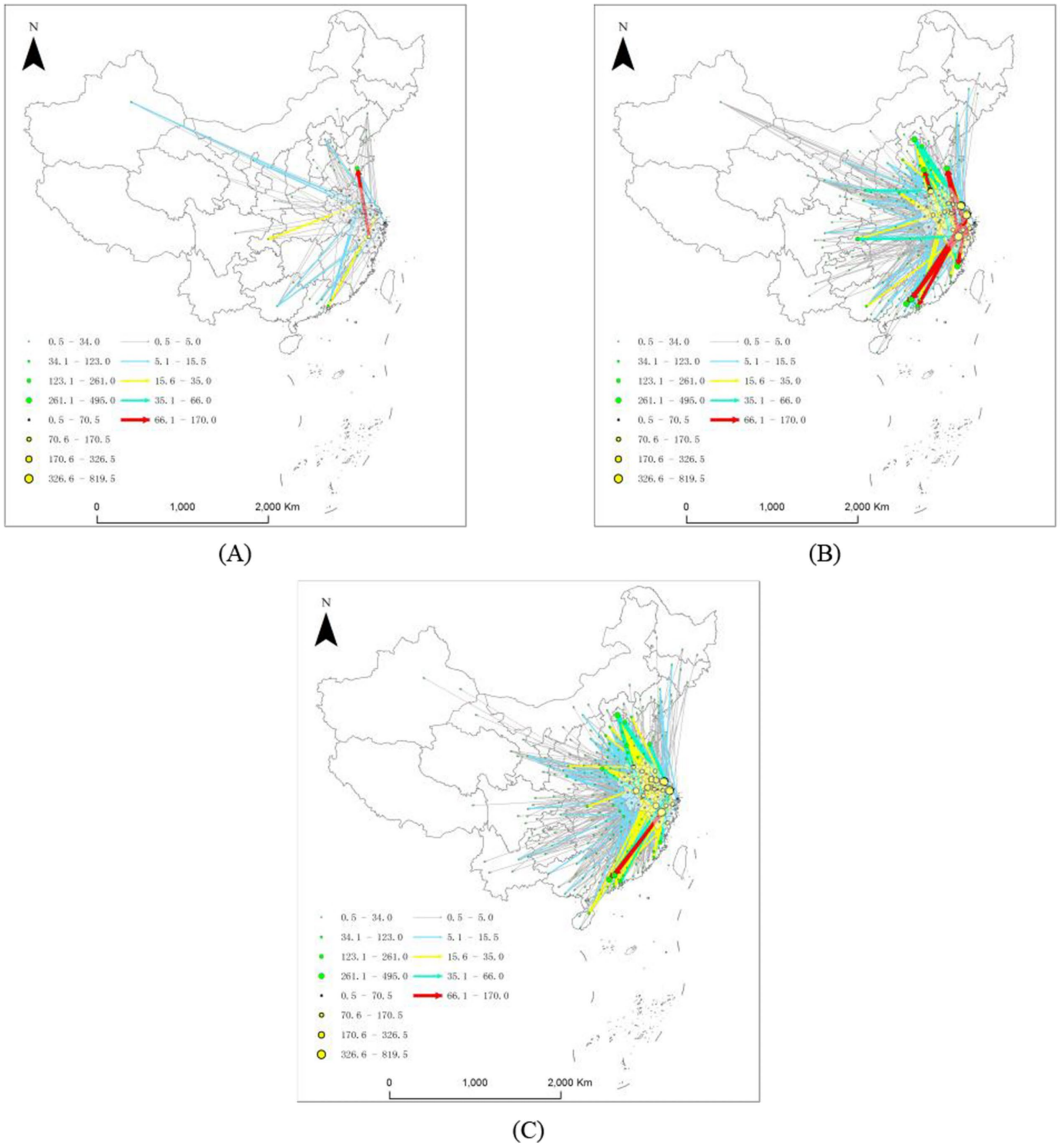
Outward pollution transfer network from the YRD. **(A)** 2012–2015; **(B)** 2016–2019; **(C)** 2020–2023.

Using the natural breakpoint method, which is based on the weights of the third–stage edges and the weighted centrality of cities, the pollution linkages were categorized into five tiers and four tiers (Figure 2). It is evident that cities in the YRD that transfer pollution outward cover most cities nationwide and exhibit a distinct core–periphery structure. This structure focuses on core YRD cities such as Shanghai, Hangzhou, and Nanjing as pollution export sources, with polluting cities primarily distributed across the Pearl River Delta, Beijing–Tianjin–Hebei region, Chengdu–Chongqing urban cluster, and numerous third– and fourth–tier cities. With respect to the pollution transfer intensity, medium– to high–intensity transfers are concentrated in economically developed or industrially concentrated regions, such as Guangzhou, Shenzhen, Foshan, and Dongguan. Extremely high–intensity transfers are concentrated in a few key routes, such as Shanghai–Beijing, Shanghai–Shenzhen, Shanghai–Guangzhou, Jinhua–Foshan, and Nantong–Beijing, indicating frequent and significant pollution transfer activities along these pathways.

The average distance of pollution transfers from the YRD is approximately 942 km, which exhibits the characteristic “proximity transfer” typical of eastern regions. This finding aligns with conclusions from previous studies, indicating that the spatial displacement of pollution-related activities in China is primarily constrained by regional institutional and regulatory environments, rather than occurring uniformly over long distances (22). However, this study quantifies the actual spatial transfer distances between cities through network analysis, providing more direct empirical evidence. Medium–to–short–distance transfers dominate, with cities such as Nantong, Xuzhou, and Yangzhou in the YRD transferring pollution to neighboring provinces at distances mostly less than 500 km, resulting in higher weights, which account for a high proportion of the total pathways and demonstrate that geographic proximity plays a crucial role in pollution transfer. More than 30% of migration pathways are within 500 km, particularly from central and northern Jiangsu province to Shandong and Anhui provinces. Although long–distance transfers to the Pearl River Delta and central–western regions carry high weights, the majority of transfers in terms of quantity remain medium–to–short distance.

Among the cities transferring pollution out of the YRD, Shanghai, Jinhua, and Nantong consistently served as the primary sources of pollution transfer (Table 2). These three cities ranked among the top three in terms of weighted outflow intensity across all three phases, with Shanghai and Jinhua exhibiting notably higher transfer intensities than the other cities during the third phase. The transfer intensity of cities such as Hangzhou, Nanjing, Hefei, Yangzhou, and Taizhou (Jiangsu) also increased markedly over time, which suggests that both the intensity and scope of pollution transfer within the YRD are continuously expanding. With respect to the cities receiving pollution outside the YRD, Beijing, Guangzhou, Foshan, Fuzhou, Zhengzhou, Shenzhen, Qingdao, and Tianjin were the primary cities that received pollution. Beijing’s pollution reception intensity steadily increased across all three phases, reaching 495 in Phase III to become China’s largest pollution–receiving city. Guangzhou, Foshan, Fuzhou, Zhengzhou, and other cities also had markedly higher pollution reception intensities, revealing a trend where pollution transfers converge toward economically developed or industrially concentrated regions such as the Beijing–Tianjin–Hebei area, the Pearl River Delta, and central provincial capitals. Overall, both pollution outflows and inflows exhibited a pronounced “pyramid” structure: a small number of cities had the overwhelming majority of pollution transfer flows, while most cities experienced relatively low levels of either inflow or outflow. This concentration intensified in the third phase, when high–intensity pollution transfers became more concentrated, amplifying the path–dependent effects of pollution migration. These pyramid-topped cities occupy pivotal positions in industrial chains, logistics systems, and population concentration as national or regional economic hubs, structurally increasing their vulnerability to transboundary pollution exposure, which both validates and enriches Li et al.’s (30) research on pathways of environmental health risk transfer. Consequently, they often assume dual roles as both pollution recipients and bearers of governance burdens. Peripheral cities may become “regulatory basins,” absorbing polluting industries. Neglecting environmental health governance in these cities will create greater risks in the future. Moreover, the intensification of path dependency effects implies that certain cities become locked in as pollution recipients, leading to long-term, structural environmental health disadvantages. These highlight the spatial mismatch among risks, responsibilities, and capability in regional environmental governance and public health prevention.

**Table 2:** Key cities in the YRD’s outward pollution transfer network.

Phase	Top 10 cities for pollution outflow	Top 10 cities for pollution inflow
Phase I	Jinhua (103.5), Shanghai (50), Yangzhou (48.5), Nantong (36), Ningbo (15), Yancheng (13.5), Lishui (13), Changzhou (11), Taizhou (Zhejiang) (10.5), Zhoushan (9.5)	Qingdao (140), Shenzhen (64.5), Beijing (22.5), Chongqing (19.5), Nanning (18), Yinchuan (13.5), Foshan (12.5), Urumqi (10.5), Shenyang (10), Chengdu (5), Shantou (5)
Phase II	Jinhua (727), Shanghai (699), Nantong (448.5), Xuzhou (182.5), Nanjing (166), Ningbo (154), Taizhou (Zhejiang) (143.5), Changzhou (142), Hangzhou (142), Yangzhou (127.5)	Beijing (386), Qingdao (306), Guangzhou (286), Foshan (267.5), Fuzhou (265), Jinan (263.5), Shenzhen (189), Tianjin (146.5), Chongqing (144.5), Zhengzhou (98.5)
Phase III	Shanghai (819.5), Jinhua (623), Nantong (595), Hangzhou (326.5), Nanjing (246), Hefei (227), Yangzhou (199.5), Taizhou (Jiangsu) (194), Changzhou (170.5), Ningbo (157.5)	Beijing (495), Guangzhou (370.5), Foshan (332), Fuzhou (261), Zhengzhou (237.5), Shenzhen (231), Qingdao (190.5), Tianjin (185.5), Jinan (150), Linyi (123)

### 3.3 Structural characteristics of pollution transfer networks within the YRD from a local perspective

Most existing research on environmental pollution in the YRD has focused on spatial clustering or spillover effects of pollution levels and governance capacities among cities (3, 4, 11–15). This section examines the structural characteristics of pollution transfer within the YRD, with a particular emphasis on the redistribution of pollution among cities.

To further analyze the structural characteristics of the pollution transfer networks within the YRD, we isolated the intra–regional pollution transfer network from the overall network. The findings (Table 3) reveal that the number of cities involved in pollution transfer increased from 37 to 41, with all of the cities in the region participating. The number of edges, average degree, and average weighted degree increased from 98, 2.649, and 8.77 to 789, 19.244, and 199.329, respectively. The network scale continued to expand, with significant increases in the depth and breadth of pollution transfer. The network diameter, average path length, and modularity index decreased markedly, while the average clustering coefficient increased, which indicates tighter connections within the pollution transfer network and a more uniform distribution of pollution transfers among cities.

**Table 3:** Basic attributes of the pollution transfer networks within the YRD.

Statistical indicators	Phase I	Phase II	Phase III
Node	37	41	41
Edge	98	475	789
Average degree	2.649	11.585	19.244
Average weighted degree	8.77	85.11	199.329
Network diameter	6	4	3
Density	0.074	0.29	0.481
Modularity	0.506	0.256	0.147
Average clustering coefficient	0.27	0.466	0.637
Average path length	2.837	1.829	1.534

As shown in Figure 2, the spatial connectivity and complexity of pollution transfer significantly increased and reflected increasingly frequent cross–city pollution transfers and denser inter–city pollution transfer activities. The pollution transfer intensity between cities such as Shanghai, Hangzhou, Nanjing, Suzhou, and Hefei was notably higher than that between other cities, indicating highly prominent pollution transfer relationships. High–weight edges were predominantly concentrated between economically developed and industrially dense cities, suggesting that these cities serve as both major pollution exporters and important receiving destinations. From a spatial distribution perspective, core cities such as Shanghai, Nanjing, Hangzhou, Suzhou, and Hefei consistently maintained high activity within the network, with their out–degree and in–degree values significantly exceeding those of other cities across all three phases. Cities such as Nantong, Taizhou (Jiangsu), Zhoushan, and Wuhu exhibited markedly higher out–degree than in–degree in the later phase, which suggests their gradual emergence as key pollution–exporting cities within the YRD. Hefei’s out–degree (376) far exceeded its in–degree (95) in the third phase, signaling its transformation from a pollution–receiving city to an emitting city. Evolutionary trends reveal that Phase I featured sparse pollution transfer relationships with generally low weights primarily confined within provinces or between neighboring cities. In Phase II, there was a marked increase in both the volume and weight of pollution transfers, with cross–provincial and off-site transfers becoming more prevalent. In the third phase, the network further densified, with a surge in high–intensity pollution transfers. Particularly between cities such as Shanghai, Hangzhou, Nanjing, and Suzhou, the pollution transfer intensity reached its peak, which reveals that both the complexity and intensity of pollution transfer within the YRD escalated under the backdrop of regional integration.

**Figure 3:**
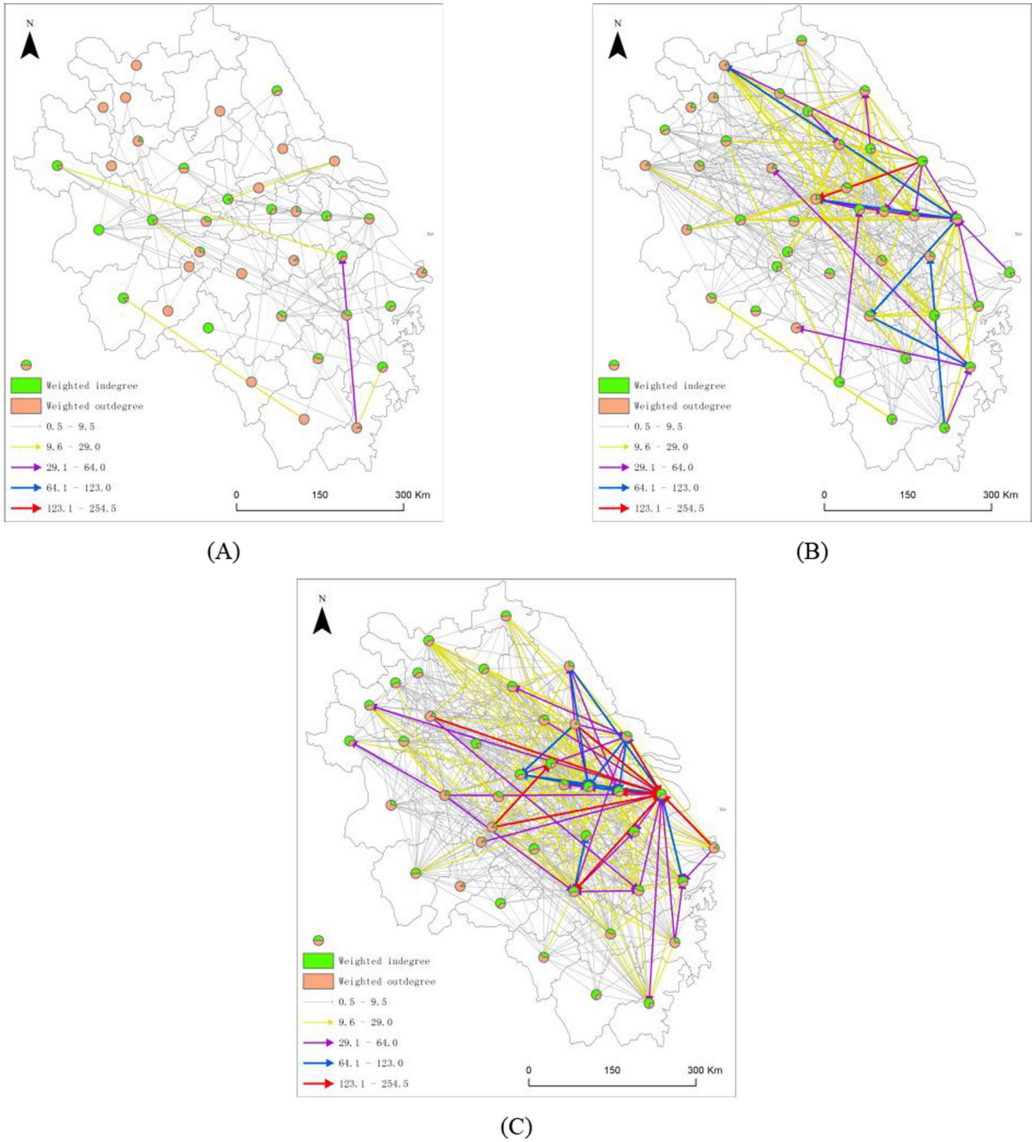
Inward pollution transfer network within the YRD. **(A)** 2012–2015; **(B)** 2016–2019; **(C)** 2020–2023.

Pollution migration in the YRD (Figure 3) exhibited a pronounced “proximity” characteristic, with an average migration distance of only 389 km, which is significantly below the national average of 942 km. Specifically, more than 40% of migration occurred within 200 km and formed a dense short–distance migration network centered on Shanghai, Nanjing, and Hangzhou. The migration distance between Shanghai and Suzhou is only 76.8 km, yet the pollution transfer intensity reached 147. The distance between Shanghai and Hangzhou is 235.6 km, with a transfer intensity of 254.5, and the highest transfer intensity occurred in the YRD. Major city pairs such as Nanjing→Hefei and Hangzhou→Ningbo maintained migration distances under 200 km but exhibited high pollution transfer intensities. This proximity characteristic likely originates from enterprises minimizing transfer costs during pollution migration, coupled with the region’s tight economic ties and coordinated policies that facilitate easier pollution shifts among neighboring cities within the YRD city cluster (61–64).

Overall, the pollution transfer network in the YRD exhibits a distinct core–periphery structure. High–intensity connections among core cities dominate the network evolution, while simultaneously driving pollution linkages with surrounding cities, revealing a pronounced “proximity” characteristic. This pronounced proximity characteristic conceals profound mechanisms of environmental health inequality. Pollution does not dissipate away from population centers but instead undergoes short-distance transfers within developed urban clusters through dense economic networks. This redistributes environmental health risks among cities with comparable economic levels but varying public health resilience. Simultaneously, low migration costs make it easier to draw smaller cities with weaker governance capabilities into high-risk networks, thereby creating regional inequalities where development is shared while risks are transferred. This motivates further analysis of the economic, institutional, technological, and public health proximity factors influencing network formation.

### 3.4 Changes in the pollution outward and inward orientation trends from a national–local perspective

**Figure 4:**
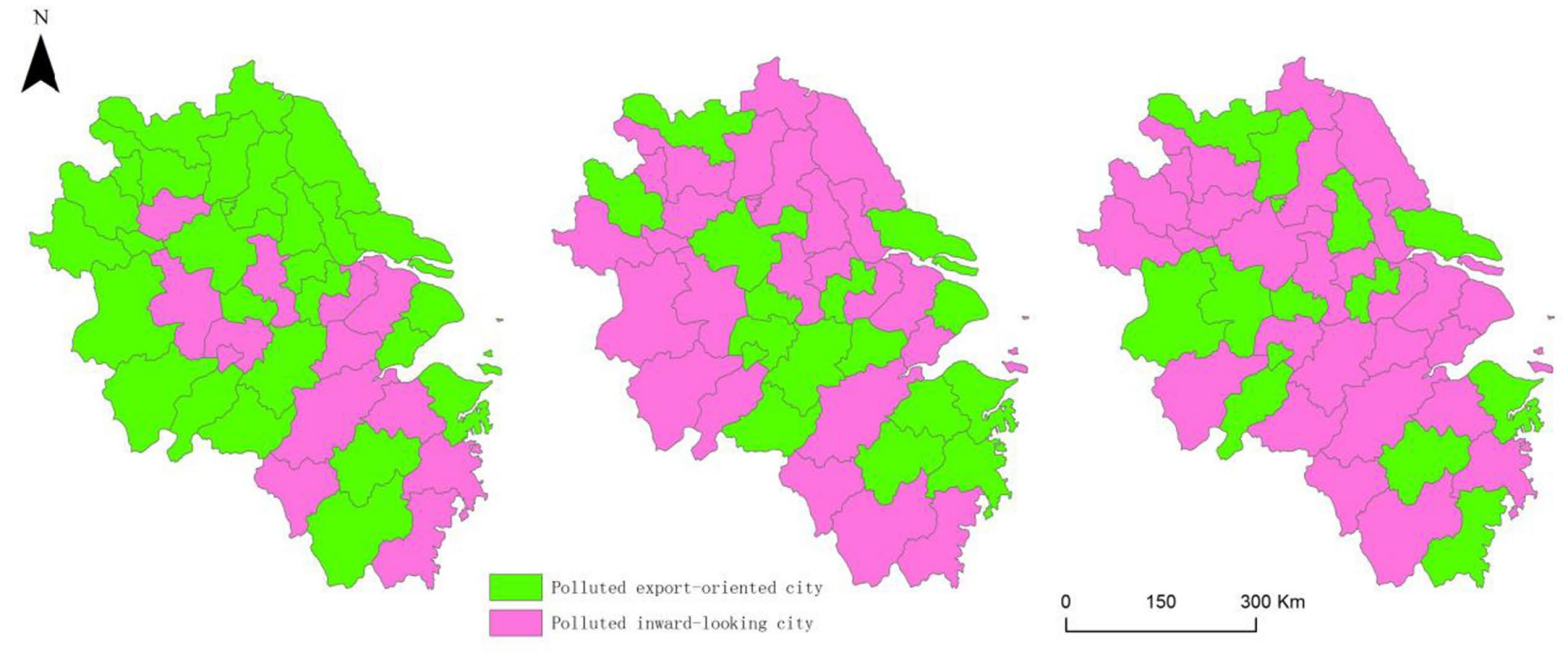
Changes in the inward and outward trends of urban pollution in the YRD.

To further elaborate on the functional role of cities within pollution transfer networks, this subsection examines the dynamic shifts in the direction of pollution outward and inward trends from both national and local perspectives. By calculating the national–local indices for cities in the YRD, the 41 prefecture–level cities were categorized into pollution outward–oriented cities and pollution inward–oriented cities (Figure 4). The number of pollution outward–oriented cities decreased from 28 in Phase I to 13 in Phase III. This indicates a reduction in the nationwide transfer of high–intensity pollution outward, whereas the pollution transfer within high–intensity areas within the YRD increased. Six cities, Jinhua, Ma’anshan, Nantong, Xuzhou, Changzhou, and Ningbo, remained polluted, exporting throughout the study period, while six others, Hangzhou, Nanjing, Bengbu, Quzhou, Suzhou, and Wuxi, consistently exhibited pollution–importing characteristics. 18 cities, including Taizhou (Jiangsu), Yancheng, and Lishui, transitioned from being pollution–exporting to pollution–importing cities, while seven cities, Tongling, Hefei, Wenzhou, Taizhou (Zhejiang), Wuhu, Shaoxing, and Huzhou, experienced the opposite shift. This trend shift suggests that, on the one hand, governmental policies in the YRD have intensified its overall environmental governance efforts, deepened regional integration, and continuously upgraded and adjusted its industrial structure. This has reduced the reliance on transferring pollution outside the region to improve local environments, with the synergistic effects of regional environmental policies beginning to emerge. On the other hand, the distribution of pollution–exporting and pollution–importing cities is closely related to their functional positioning. Central cities such as Hangzhou and Nanjing prioritize developing high–tech industries and modern services and impose high barriers to entry for high–pollution industries, thereby becoming pollution–inward cities. In contrast, resource–based or industrially robust cities such as Ma’anshan and Xuzhou, which face greater challenges in industrial transformation, retain more polluting industries and thus remain pollution–outward cities. These divergent orientation trajectories underscore the importance of differentiated governance strategies tailored to cities’ functional positions within the regional pollution transfer system. As carbon transfer recipient cities, most are industrial hubs that enjoy economic advantages without bearing commensurate carbon emission responsibilities (65). This phenomenon not only exacerbates environmental health inequalities between cities but also reinforces a spatial division of labor characterized by asymmetric patterns of pollution absorption, health risks, and benefit distribution.

## 4 Analysis of the network influencing factors

### 4.1 Selection of the influencing factors

A review of the literature on urban network influencing factors reveals that multidimensional proximity and other factors significantly impact urban networks (43–51). Therefore, guided by principles of comprehensiveness and data availability, the following variables were selected: the pollution transfer intensity between cities in Phase III’s YRD outward pollution network, the pollution transfer intensity within the YRD inward pollution transfer network, and the pollution transfer intensity across the entire YRD pollution transfer network. These serve as the dependent variables. The independent variables include the geographic distance between cities (representing geographic proximity), the absolute difference in GDP between cities (representing economic proximity), the absolute difference in the tertiary industry output share of GDP between cities (representing industrial proximity), the absolute difference in the number of physicians per 10,000 people (representing public health proximity), the absolute difference in the number of patent authorizations between cities (representing technological proximity), and the absolute difference in the administrative tier. Drawing on existing research that operationalizes administrative hierarchy as a sequential institutional attribute (66), which reflects disparities in urban governance capacity and policy access, this study assigns administrative tiers as follows: municipalities directly under the central government (4), sub-provincial cities (3), provincial capitals (2), and prefecture-level cities (1). Institutional proximity is measured by the absolute difference in these values. Physicians per 10,000 people are used as a proxy for health system capacity and population health resilience. Differences in this indicator reflect variation in cities’ ability to withstand pollution shocks. Before the geographic probe analysis, a natural breakpoint analysis was conducted using ArcGIS to classify the data into four levels. The geographic distances between cities were calculated by ArcGIS based on city coordinates. The data on GDP, tertiary industry output share of GDP, patent grants, and physicians per 10,000 population were sourced from the Statistical Yearbook of Chinese Cities.

### 4.2 Analysis of the influencing factors

A Geodetector analysis reveals that the proximity factors across different dimensions strongly influence the pollution transfer networks within the YRD (the *p* values are significant at the 1% level). However, their intensity and dominant domains exhibit distinct variations.

Our findings (Table 4) highlight that pollution transfer in the YRD operates as a complex adaptive system, where multidimensional proximities interact in nonlinear ways to shape network evolution. With respect to the single–factor effects, economic proximity is the core driver of intra–regional pollution transfer in the YRD (*Q* value = 0.161), indicating that tight economic ties between cities are the most potent engine for pollution movement within the region. Institutional proximity (*Q* value = 0.142) ranks second and suggests that the similarities in institutional frameworks between cities create a “soft environment” conducive to pollution transfer. Regions with minimal institutional differences face lower regulatory cost disparities among enterprises, resulting in reduced barriers and risks for pollution transfer. Concerning pollution transfer out of the YRD, institutional proximity and technological proximity emerge as significant factors. For enterprises to successfully relocate pollution beyond the region, the primary condition is identifying the destinations with institutional environments and technological absorption capacities comparable to those within the YRD. At the aggregate level of pollution transfer from the YRD, economic factors again demonstrate the strongest dominance.

**Table 4:** Geodetector factor detection results.

Proximity	Outward pollution transfer		Intra–regional pollution transfer		Overall pollution transfer	
	*Q* value	*p*-value	*Q* value	*p*-value	*Q* value	*p*-value
Geographical proximity	0.011	0.007	0.031	0.000	0.025	0.000
Economic proximity	0.027	0.000	0.161	0.000	0.065	0.000
Industrial proximity	0.025	0.000	0.098	0.000	0.031	0.000
Technological proximity	0.031	0.000	0.084	0.000	0.044	0.000
Institutional proximity	0.034	0.000	0.142	0.000	0.050	0.000
Public health proximity	0.022	0.000	0.020	0.000	0.005	0.038

Additionally, the influence of public health proximity exhibits distinct spatial directionality. Its explanatory power is stronger for outward pollution transfer than for intra–regional transfer, which indicates that the similarities in public health standards among cities play a relatively more important role in the process of transferring pollution outside the YRD. This result may originate from the “pollution refuge” effect, where polluting enterprises tend to relocate from regions with high public health standards and strict regulation to surrounding areas with comparable health levels but greater development pressures and relatively lower entry barriers. However, the explanatory power of public health proximity is weakest for overall pollution transfer, suggesting that when outward and inward transfers are not distinguished, its independent effect is overshadowed by other stronger factors. Notably, the Q values for geographic proximity are consistently the lowest; this signals that although physical distance significantly influences pollution transfer, it is not the primary barrier.

Further analysis (Table 5) using factor interaction detection reveals that the Q values for all pairwise interactions between factors are significantly greater than the independent contributions of any single factor, demonstrating a universal “two–factor enhancement” or “nonlinear enhancement” effect. This finding indicates that pollution transfer in the YRD cannot be explained by a single factor. Geographical proximity, as a foundational factor, significantly enhances the explanatory power when it interacts with any other proximity dimension (economic, industrial, technological, or institutional), particularly in intra–regional transfers (e.g., geographical proximity ∩ institutional proximity, *Q* value = 0.247). This primarily stems from the positive moderating effect of geographic proximity: reduced physical distance provides the most fundamental condition for pollution transfer, and other proximity dimensions are amplified when geographic proximity is present (60, 63, 64, 67, 68). The most potent interaction combination emerges between economic proximity and institutional proximity (intra–regional transfer *Q* = 0.284) to form a “golden pathway” for pollution transfer. Strong economic ties provide both the motivation and channels for pollution transfer, and similar institutional environments reduce transaction costs and policy risks, which facilitate smoother pollution shifts. Although public health proximity has a relatively weak independent contribution, it has strong synergistic effects when it interacts with other proximity factors. Following the interaction with economic proximity in intra–regional pollution transfer, its explanatory power increases to 0.273 and becomes the second–largest interaction term after economic proximity ∩ and institutional proximity. This outcome suggests that pollution transfer also follows paths aligned with similar levels of public health, with increased transfer occurring between cities that share a similar public health foundation, which demonstrates that environmental health inequalities do not stem from disparities in public health capacity but are rooted in regional economic and power structures. Cities with similar health foundations experience differentiated risk exposures due to varying bargaining positions within regional divisions of labor. This leads to inequalities characterized by homogeneous starting points and heterogeneous outcomes, eroding regional health equity.

**Table 5:** Geodetector interactive detection results.

Interaction item	Outward pollution transfer		Intra–regional pollution transfer		Overall pollution transfer	
	*Q* value	Interaction type	*Q* value	Interaction type	*Q* value	Interaction type
Geographical proximity ∩ economic proximity	0.053	Nonlinear enhancement	0.242	Nonlinear enhancement	0.138	Nonlinear enhancement
Geographical proximity ∩ industrial proximity	0.054	Nonlinear enhancement	0.183	Nonlinear enhancement	0.099	Nonlinear enhancement
Geographical proximity ∩ technological proximity	0.051	Nonlinear enhancement	0.162	Nonlinear enhancement	0.100	Nonlinear enhancement
Geographical proximity ∩ institutional proximity	0.050	Nonlinear enhancement	0.247	Nonlinear enhancement	0.128	Nonlinear enhancement
Geographical proximity ∩ public health proximity	0.046	Nonlinear enhancement	0.071	Nonlinear enhancement	0.042	Nonlinear enhancement
Economic proximity ∩ industrial proximity	0.047	Dual–factor enhancement	0.178	Dual–factor enhancement	0.073	Dual–factor enhancement
Economic proximity ∩ technological proximity	0.078	Nonlinear enhancement	0.242	Dual–factor enhancement	0.127	Nonlinear enhancement
Economic proximity ∩ institutional proximity	0.063	Nonlinear enhancement	0.284	Dual–factor enhancement	0.103	Dual–factor enhancement
Economic proximity ∩ public health proximity	0.057	Nonlinear enhancement	0.273	Nonlinear enhancement	0.105	Nonlinear enhancement
Industrial proximity ∩ technological proximity	0.075	Nonlinear enhancement	0.138	Dual–factor enhancement	0.057	Dual–factor enhancement
Industrial proximity ∩ institutional proximity	0.047	Dual–factor enhancement	0.169	Dual–factor enhancement	0.064	Dual–factor enhancement
Industrial proximity ∩ public health proximity	0.049	Dual–factor enhancement	0.163	Nonlinear enhancement	0.043	Nonlinear enhancement
Technological proximity ∩ institutional proximity	0.097	Nonlinear enhancement	0.199	Dual–factor enhancement	0.114	Nonlinear enhancement
Technological proximity ∩ public health proximity	0.069	Nonlinear enhancement	0.149	Nonlinear enhancement	0.073	Nonlinear enhancement
Institutional proximity ∩ public health proximity	0.065	Nonlinear enhancement	0.207	Nonlinear enhancement	0.061	Nonlinear enhancement

Accordingly, pollution transfer in the YRD is not driven by a single factor but represents a complex network phenomenon dominated by economic proximity linked to institutional proximity and grounded in geographic proximity. An analysis that incorporates public health proximity reveals that similar regional public health levels, although they exhibit weak independent effects, form highly intensive interactions with economic and institutional proximity and emerge as a crucial synergistic factor that influences pollution transfer networks.

## 5 Conclusion and implications

### 5.1 Conclusion

In this study, social network analysis and the geographical detector method are employed to construct a systematic pollution transfer network for the YRD using environmental penalty data from large-scale enterprises over the past decade. From both national and local perspectives, this study examines the structural characteristics of pollution transfer and its underlying multidimensional proximity mechanisms. The main conclusions are summarized as follows:

1. The pollution transfer network in the YRD has continued to expand over time, with increasingly dense inter-city linkages forming a pronounced core–periphery structure centered on Shanghai. This structure facilitates the outward diffusion of pollution-related environmental health risks from core cities to peripheral regions and further to the national scale.
2. In terms of spatial scope and intensity, intra-regional transfers within the YRD remain significantly stronger than extra-regional transfers. Meanwhile, the outward pollution transfer network has expanded markedly toward major urban agglomerations such as the Pearl River Delta, the Beijing–Tianjin–Hebei region, and the Chengdu–Chongqing urban cluster, indicating an ongoing redistribution of environmental health risks across major population centers.
3. From a distance perspective, pollution transfers are characterized by strong spatial proximity. The average intra-regional transfer distance is only 389 km, highlighting the dominance of short-distance transfers and suggesting that neighboring cities with close socioeconomic linkages may experience compounded environmental health pressures.
4. Regarding driving mechanisms, multidimensional proximity jointly shapes pollution transfer patterns. Economic proximity dominates intra-regional transfers, while institutional and technological proximity play more significant roles in extra-regional transfers. Public health proximity shows limited independent explanatory power but significantly enhances pollution transfer when interacting with economic and institutional factors, indicating that combined proximity effects outweigh single-factor influences.

### 5.2 Implications and recommendations

The findings of this study carry important implications for public health governance and regional health equity.

At the regional governance level, the analysis reveals that pollution tends to shift between cities with similar public health levels. The YRD should establish a regional public health and environmental risk early warning platform, integrate urban public health into regional industrial planning and environmental risk assessment systems, and create a joint prevention and control mechanism for regional health risks. By integrating environmental monitoring and public health data, real–time monitoring of the potential impacts of pollution transfer on urban public health can prevent pollution from shifting to cities with comparable public health foundations but weaker regulatory oversight.

From an inter-city regulatory perspective, regions should promote the inter–city sharing of environmental penalty information to enable real–time tracking and joint disciplinary actions against enterprises’ off-site environmental violations. High–intensity pollution transfer pathways require intensive monitoring complemented by a “blacklist” system to restrict high–polluting enterprises from relocating to ecologically sensitive or low–carrying capacity areas.

At the city-specific level, differential environmental governance strategies should be implemented in the YRD to manage key cities precisely. Environmental accountability tracing should be strengthened for the core cities that export pollution within the transfer network, and the core cities that receive pollution should increase the carrying capacity and risk resilience of their environmental infrastructure.

### 5.3 Theoretical contributions

This study contributes to public health and environmental health research in three main ways.

Specifically, an analytical framework grounded in a dual national–local perspective reveals the spatiotemporal dynamics of pollution transfer through a comparative analysis of network indicators across scales. This approach overcomes the limitations of single–scale research and establishes a new hierarchical paradigm for urban network studies (29–41).

In addition, by leveraging the big data on off-site environmental penalties imposed on enterprises, this study overcomes the underestimation inherent in traditional pollution transfer research based on transfer estimates and offers a new empirical foundation for pollution transfer analysis (20–28).

Moreover, by introducing multidimensional proximity theory, this study systematically reveals the interactive mechanisms of economic, institutional, technological, geographic, industrial, and public health proximity in terms of pollution transfer, providing theoretical foundations and policy references for pollution control in the YRD and nationwide (42–50).

### 5.4 Research limitations and future directions

Although pollution penalty records provide confirmed evidence of pollution transfer, they may not fully capture informal, unreported, or unpenalized transfer activities. Consequently, the constructed network reflects only the observable and regulated component of pollution transfer, not its full spectrum.

Furthermore, this study primarily measures public health risks and environmental health inequalities through public health service capacity indicators, a choice driven by considerations of data availability and comparability. These indicators reflect response capabilities rather than actual health outcomes. Future research could integrate outcome-based health data to more directly capture the health impacts of pollution transfer.

Additionally, this study adopts prefecture-level cities as the unit of analysis. It primarily examines inter-city pollution transfer relationships, without fully exploring micro-level behavioral mechanisms at the enterprise level. Moreover, the analysis focuses on pollution transfer within the YRD and its linkages with the national level, without systematically incorporating interregional transfer dynamics among other regions. Future research could integrate enterprise-level behavioral data with multi-scale spatial analysis and expand the geographic scope to more comprehensively assess regional roles and positioning within the national pollution transfer system.

Finally, this study’s timeframe concludes in 2023. While this period encompasses key policy transitions and stable structural trends, future research may incorporate more recent data and employ finer-grained time windows to examine emerging dynamics within an evolving regulatory and economic landscape.

## Data Availability

The raw data supporting the conclusions of this article will be made available by the authors, without undue reservation.

## Footnotes

1 https://www.mee.gov.cn/ywgz/sthjzf/zfzdyxzcf/202407/t20240730_1082928.shtml. https://www.mee.gov.cn/ywgz/sthjzf/zfzdyxzcf/202411/t20241125_1096570.shtml. https://www.mee.gov.cn/ywgz/sthjzf/zfzdyxzcf/202311/t20231116_1056587.shtml.
